# Gender differences and access to a sports dietitian influence dietary habits of collegiate athletes

**DOI:** 10.1186/s12970-016-0149-4

**Published:** 2016-10-18

**Authors:** Michael V. Hull, Andrew R. Jagim, Jonathan M. Oliver, Mike Greenwood, Deanna R. Busteed, Margaret T. Jones

**Affiliations:** 1 Center for Sports Performance, George Mason University, Fairfax, VA USA; 2 Exercise & Sport Science Department, University of Wisconsin – La Crosse, La Crosse, WI USA; 3 Exercise & Sport Performance Laboratory, Kinesiology Department, Texas Christian University, Fort Worth, TX USA; 4 Exercise & Sport Nutrition Laboratory, Texas A&M University, College Station, TX USA; 5 Division of Health & Human Performance, George Mason University, 10890 George Mason Circle, MS 4E5, Manassas, VA 20110-2203 USA

**Keywords:** Nutritional supplementation, Dietary behaviors, NCAA student athlete, Nutrient periodization, Survey

## Abstract

**Background:**

Limited research exists on the effect of a sports dietitian (SD) on athletes’ dietary habits and nutrient periodization, which is the deliberate manipulation of macronutrient intake to match training goals. Further, the difference in dietary habits between men and women collegiate athletes has been understudied. A survey questionnaire examining dietary habits and practices was administered to athletes at two universities that employed a full time SD. Not all athletes used the SD as their primary source for nutritional guidance. The purposes were to examine the effect of a SD as a primary source of nutrition information, and the effect of gender on dietary habits in collegiate athletes.

**Methods:**

Three hundred eighty-three women (*n* = 240) and men (*n* = 143) student-athletes (mean ± SD: age = 19.7 ± 1.4 years) from 10 collegiate sports took a 15-min survey consisting of questions on dietary habits and practices. Topics queried included eating habits, breakfast habits, hydration habits, nutritional supplementation use, pre-workout nutrition, post-workout nutrition, nutrition during team trips, and nutrient timing. Data were sorted by the athlete’s source of nutritional information (i.e., sport dietitian, other). Data analysis consisted of descriptive statistics and 2-way Pearson X^2^ analyses (*p* ≤ 0.10).

**Results:**

When a SD was indicated as the primary nutrition information source, athletes appeared to have a greater understanding of nutrient periodization (47.12 % vs. 32.85 %), were more likely to have school-provided boxed meals while on team trips (21.29 % vs. 6.77 %), and also less likely to consume fast food while on team trips (9.90 % vs. 19.55 %). Men athletes consumed fast food or restaurant meals more frequently, had higher weekly and more frequent alcohol intake during the competitive season. Women athletes were more likely to prepare meals, eat breakfast 7 days a week, and have school-provided boxed meals.

**Conclusions:**

Positive effects on dietary habits were observed when a SD was the primary nutrition information source. Practitioners should be aware of the gender differences in alcohol intake, fast food consumption, and knowledge of nutrient periodization. Collegiate athletes and athletic staff members could benefit from SD access to safeguard against dietary habits detrimental to performance.

## Background

Nutrition education by a registered dietitian (RD) or a registered dietitian nutritionist (RDN) has been shown to improve the dietary knowledge of collegiate athletes and the general public [1–3]. Sports dietitians (SD) are those RD/RDNs who have achieved additional credentialing such as becoming a Board Certified Specialist in Sports Dietetics (CSSD) from the Academy of Nutrition and Dietetics. Thus, SDs may be able to provide more robust and comprehensive sports nutrition education and counseling. Sound dietary practices can have a profound impact upon the athlete’s health and performance [4, 5]. Not only does the National Collegiate Athletic Association (NCAA) restrict the amount and types of food that can be provided to athletes [6], but also collegiate athletic departments are often limited in terms of nutrition staff and resources, thereby restricting services provided to the student-athletes. The first full-time RD was hired by a collegiate athletic department in 1994, to implement nutrition programs for the athletic teams [7], but the CSSD credentialing did not become available until 2006 [8]. Currently, there are 88 full-time SDs working within 61 schools in major college conferences in the United States and many are the sole SD at their respective schools, servicing anywhere from 350 to 600 student athletes [9]. Although progressive in nature, this represents only 5.4 % of the NCAA member schools throughout the United States [10]. In 2005, the Certified Sports Nutritionist from the International Society of Sports Nutrition (CISSN) became available [Jose Antonio, personal communication, May 6, 2016]. The CISSN is a professional accreditation that requires practitioners to possess a basic knowledge of sports nutrition without necessarily holding the RD/RDN credential. Typically, smaller and minimally funded colleges and universities at the Division II and III level do not have the ability or means to offer sport nutrition programs or services from an RD. As a result, they may rely on practitioners who have other primary roles (e.g., athletic trainer, strength coach) to provide nutritional guidance [11]. Furthermore, a large evidence base now exists that supports the need for athletes to employ specific dietary strategies to achieve peak athletic performance [4, 5]. The NCAA student-athletes are continuously exposed to periods of intense training, conditioning, and practice and; therefore, would likely benefit from well-defined, nutrition practices in order to reduce training-related injuries and optimize sports performance.

A recent review by Heaney et al. [12], which examined the nutrition knowledge level of collegiate, high school, and recreational athletes, found comparable scores between the athletes and non-athlete controls. When examining the relationship between athletes’ nutritional knowledge and dietary habits, a weak positive association was observed (*r* < 0.44) [12]. While athletes may possess moderate general nutrition knowledge, the majority appears to have limited knowledge of best performance nutrition practices [11, 13, 14] and to have difficulty applying that knowledge into consistent dietary habits [14, 15]. Subsequently, many athletes report feeling regret over consuming foods with little nutritional value, not fueling sufficiently for practice and competition, and their overall food choices [16].

While student-athlete desire for access to a SD is high [17, 18], few data are available that examine the impact such contact may have upon the dietary habits of athletes. Prior studies have indicated the need to expand upon this line of research [1, 11, 12], and there are currently limited data on whether or not access to a SD can impact the nutrition knowledge and dietary habits of student-athletes [17]. Published research with NCAA women volleyball athletes has shown that educational sessions with a RD can improve both nutritional knowledge and dietary intake [1]. Additionally, when examining gender differences in dietary and nutrition knowledge, results are largely equivocal between the genders [12] with some reporting better results for women [17, 18]. In regard to dietary habits, prior research has mainly focused upon the assessment of solely men or solely women without making gender comparisons [13, 14, 19–23]. Among studies that have examined gender differences in collegiate athletes [24–26], all focused primarily upon dietary intake patterns assessed using methods such as 1- or 3- day food recalls.

The purpose of the current study was to gain a more comprehensive view of dietary habit differences in NCAA Division I athletes by assessing a broad spectrum of nutritional practices, ranging from nutrient periodization, which is the deliberate manipulation of macronutrient intake to match training goals and enhance performance [27], to pre-workout and post-workout dietary habits. The first aim was to examine the dietary habits and dietary behaviors of collegiate athletes who had access to a full time SD as their primary nutrition information source and compare them to those who used non-SD sources. The second aim was to assess differences in dietary habits and practices between men and women collegiate athletes.

## Methods

### Overview

This descriptive research study employed a cross-sectional survey designed to assess dietary habits and nutrient periodization practices in NCAA Division I athletes. A survey questionnaire was designed in a similar fashion to one previously used to assess the dietary eating habits of NCAA Division III athletes [28]. The use of the current survey allowed for the assessment of specific nutrition habits, which included hydration and supplement use, as well as nutrient periodization strategies across two institutions and ten sports at the NCAA Division I level.

### Subjects

A total of 383 NCAA Division I athletes (240 women, 143 men, mean ± SD: age = 19.7 ± 1.4 years) who represented ten intercollegiate sports from two universities (University 1, *n* = 278; University 2, *n* = 105) in two athletic conferences (i.e., Atlantic 10, Atlantic Coast Conference) completed the survey. All athletes were medically cleared for intercollegiate athletic participation, had the investigative procedures explained to them beforehand, and signed an institutionally approved consent form to participate. The Institutional Review Board for Human Subjects at each participating institution approved all procedures. All subjects were 18 years of age or older and had the opportunity to work with a SD if they chose to do so. Subject descriptive data are detailed in Table 1.

**Table 1:** Descriptive Statistics for Survey Respondents (*n* = 383)

Athletes working with a sports dietitian		60.10 %
Athletes not working with a sports dietitian		39.90 %
Sport Played	Basketball	6.80 %
	Golf	4.20 %
	Lacrosse	6.30 %
	Rowing	10.70 %
	Soccer	6.50 %
	Soccer	6.80 %
	Softball	12.50 %
	Tennis	2.90 %
	Track/Field	21.90 %
	Volleyball	14.90 %
	Wrestling	6.50 %

### Sports dietitian

Both institutions employed a full-time SD who had similar foundational training and credentialing. Professional responsibilities of the SD’s included working with individual athletes, teams, coaches, and other athletic staff members to provide sports nutrition services, which included nutrition education and counseling with the primary aim being that of improving athlete performance. Information was also distributed through general outreach efforts by the SD, such as educational bulletin boards, athletics/sports nutrition website content, social media, sports nutrition lectures, and informal interaction with the athletes and athletic staff.

### Procedures

The authors designed the survey questionnaire to assess the dietary habits of NCAA-Division I athletes based upon one previously used to assess the dietary eating habits of NCAA Division III athletes [28]. Procedures were followed to establish content validity. Initially, a qualitative researcher with content knowledge of sport nutrition reviewed the survey. Suggestions regarding placement and wording of certain questions were incorporated into the second version. Next, two SDs from the two participating institutions reviewed the instrument. Suggestions that were added into the third version included a separate section on hydration and representative pictures of serving sizes. Finally, the survey was piloted with a group (*n* = 6) of athletic trainers, graduate assistant coaches, and strength coaches from the involved universities. Suggestions provided by the aforementioned group related primarily to phrasing of specific questions, and were taken into account during the completion of the fourth version, which was the final draft. No participants in the pilot study served as subjects in the current study.

The survey questionnaire consisted of 62 total questions distributed over nine sections. The nine sections were represented in the following order: sport participation, general eating habits, breakfast, hydration, nutritional supplements, post-workout nutrition, nutrition during team trips, nutrient periodization, and demographic information. There were 25 closed-ended, 22 interval, 7 multiple choice, and 8 open-ended questions. Open-ended questions were asked in regard to: demographics (*n* = 2), eating habit changes with training season (*n* = 2), listing of breakfast foods (*n* = 1), listing of supplements currently using (*n* = 1), and sports participation (*n* = 2).

### Data collection

The same researchers administered the survey questionnaires during scheduled testing sessions. First, athletes read and signed the informed consent form. Next, researchers reviewed the survey’s instructions with the subjects, and remained throughout the testing sessions in order to answer questions. Athletes were provided a pencil and a survey, sat apart from each other, and no talking or leaving their seats was permitted. Upon completion, subjects placed the survey questionnaire into an envelope. All survey questionnaires were anonymous. The only identifying information consisted of age, gender, sport, and university name. Sport coaches were not present during data collection. There was no time limit and the total time for completion of the survey ranged from 15–20 min depending upon the individual.

### Data analysis

The data were analyzed to present descriptive data sorted by the athlete’s source of nutritional information (i.e., SD, other), and by gender. Data analysis consisted of descriptive statistics and 2-way Pearson X^2^ analyses (*p* ≤ 0.10). All data were analyzed using SPSS V.22 (IBM Corporation; Armonk, NY).

## Results

Both institutions employed a full time SD. Of the 383 (240 women, 143 men) total survey respondents, 348 answered the item in regard to “who was in charge of implementing/directing their sport dietary plan” and 35 left the item unanswered. Of the 348 respondents, 209 or 60.10 % reported working with a SD for the purpose of dietary planning (AthSD) while 139 or 39.90 % of athletes chose not to meet with the SD (AthNoSD) (Table 1).

### AthSD vs. AthNoSD

The AthSD group was more likely than the AthNoSD group to have post-workout nutrition options (Table 2) available (60.50 % vs. 40.60 %, *X* ^2^ = 13.784, *p* = 0.001). The primary protein source following physical activity for the AthSD group was more likely to be chicken (62.50 % vs. 49.28 %, *X* ^2^ = 6.678, *p* = 0.035). School-provided boxed breakfast, lunch, or dinner to the athletes while on team trips (Table 3) was more likely to occur with AthSD group (21.29 % vs. 6.77 %, *X* ^2^ = 13.11, *p* = 0.001) due to decisions made by the SD. Athletes from the AthSD group were less likely to consume fast food prior to practice or competition while on team trips (9.90 % vs. 19.55 %, *X* ^2^ = 6.477, *p* = 0.039). The survey defined fast food as food that can be prepared and served quickly without prior seating by a server (e.g., McDonald’ s, Burger King, Dunkin Donuts). The AthSD group appeared to have a better awareness of the need to periodize nutrient intake (Table 4) by adjusting caloric intake according to demands of the training cycle. The AthSD group indicated they did not consume the same amount of daily calories during the off-season and in-season (47.12 % vs. 32.85 %, *X* ^2^ = 9.973, *p* = 0.041).

**Table 2:** Post-Workout Nutrition

Question	Response	AthSD		AthNoSD		Unanswered		Total		
		N	%	N	%	N	%	N	%	Pearson X^2^ and *p* value
Does your athletic department/team provide post-workout nutrition options	Yes	121	60.50	54	40.60	0	0.00	175	52.40	13.784
	No	79	39.50	79	59.40	1	100.00	159	47.60	0.001
	Total	200	100.00	133	100.00	1	100.00	334	100.00	
		N	%	N	%	N	%	N	%	Pearson X^2^ and *p* value
What is the primary source of protein you eat most following practice/training/competition - Selected for “Chicken”	Selected	130	62.50	68	49.28	1	100.00	199	57.35	6.678 0.035
	Not Selected	78	37.50	70	50.72	0	0.00	148	42.65	
	Total	208	100.00	138	100.00	1	100.00	347	100.00	
		N	%	N	%	N	%	N	%	Pearson X^2^ and *p* value
What is the primary source of protein you eat most following practice/training/competition - Selected for “Eggs”	Selected	37	17.79	37	26.81	1	100.00	75	21.61	7.624 0.022
	Not Selected	171	82.21	101	73.19	0	0.00	272	78.39	
	Total	208	100.00	138	100.00	1	100.00	347	100.00	
		N	%	N	%	N	%	N	%	Pearson X^2^ and *p* value
What is the primary source of carbohydrates you eat most following practice/training/competition - Selected for “I do not know”	Selected	14	6.73	7	5.07	1	100.00	22	6.34	15.200 0.001
	Not Selected	194	93.27	131	94.93	0	0.00	325	93.66	
	Total	208	100.00	138	100.00	1	100.00	347	100.00	
Question	Response	Men		Women		Total				
		N	%	N	%	N	%			Pearson X^2^ and *p* value
Does your athletic department/team provide post-workout nutrition options	Yes	61	45.19	132	57.14	193	52.73			4.888
	No	74	54.81	99	42.86	173	47.27			0.027
	Total	135	100.00	231	100.00	366	100.00			
		N	%	N	%	N	%			Pearson X^2^ and *p* value
What is the primary source of protein you eat most following practice/training/competition - Selected for “Burger/Steak”	Selected	24	16.7	19	7.95	43	11.26			6.989 0.008
	Not Selected	119	83.22	220	92.05	339	88.74			
	Total	143	100.00	239	100.00	382	100.00			
		N	%	N	%	N	%			Pearson X^2^ and *p* value
What is the primary source of carbohydrates you eat most following practice/training/competition - Selected for “Rice/Pasta”	Selected	99	69.23	126	52.72	225	58.90			10.076 0.002
	Not Selected	44	30.77	113	47.28	157	41.10			
	Total	143	100.00	239	100.00	382	100.00			
		N	%	N	%	N	%			Pearson X^2^ and *p* value
What is the primary source of carbohydrates you eat most following practice/training/competition - Selected for “Grab-and-Go Bar”	Selected	8	5.59	39	16.32	47	12.30			9.535 0.002
	Not Selected	135	94.41	200	83.68	335	87.70			
	Total	143	100.00	239	100.00	382	100.00			
		N	%	N	%	N	%			Pearson X^2^ and *p* value
What is the primary source of carbohydrates you eat most following practice/training/competition – Selected for “Fruit Juice/Whole Fruit”	Selected	33	23.08	81	33.89	114	29.84			4.998 0.025
	Not Selected	110	76.92	158	66.11	268	70.16			
	Total	143	100.00	239	100.00	382	100.00

**Table 3:** Nutrition During Team Trips

Question	Response	AthSD		AthNoSD		Unanswered		Total		
		N	%	N	%	N	%	N	%	Pearson X^2^ and *p* value
What type of food is provided for you prior to practice/competition when on the road - Selected for “Fast Food”	Selected	20	9.90	26	19.55	0	0.00	46	13.69	6.477 0.039
	Not Selected	182	90.10	107	80.45	1	100.00	290	86.31	
	Total	202	100.00	133	100.00	1	100.00	336	100.00	
		N	%	N	%	N	%	N	%	Pearson X^2^ and *p* value
What type of food is provided for you prior to practice/competition when on the road - Selected for “School provided Boxed Breakfast/Lunch/Dinner”	Selected	43	21.29	9	6.77	0	0.00	52	15.48	13.110 0.001
	Not Selected	159	78.71	124	93.23	1	100.00	284	84.52	
	Total	202	100.00	133	100.00	1	100.00	336	100.00	
Question	Response	Men		Women		Total				
		N	%	N	%	N	%			Pearson X^2^ and *p* value
What type of food is provided for you prior to practice/competition when on the road - Selected for “Fast Food”	Selected	27	19.85	23	9.91	50	13.59			7.214 0.007
	Not Selected	109	80.15	209	90.09	318	86.41			
	Total	136	100.00	232	100.00	368	100.00			
		N	%	N	%	N	%			Pearson X^2^ and *p* value
What type of food is provided for you prior to practice/competition when on the road - Selected for “School provided Boxed Breakfast/Lunch/Dinner”	Selected	12	8.82	42	18.10	54	14.67			5.897 0.015
	Not Selected	124	91.18	190	81.90	314	85.33			
	Total	136	100.00	232	100.00	368	100.00			
		N	%	N	%	N	%			Pearson X^2^ and *p* value
Is food available directly after games or between same-day competitions	Yes	110	79.71	209	90.48	319	86.45			12.370 0.002
	No	9	6.52	13	5.63	22	5.96			
	N/A	19	13.77	9	3.90	28	7.59			
	Total	138	100.00	231	100.00	369	100.00

**Table 4:** Nutrient Periodization

Question	Response	AthSD		AthNoSD		Unanswered		Total		
		N	%	N	%	N	%	N	%	Pearson X^2^ and *p* value
Do you eat the same amount of daily calories during off-season and in-season	Yes	46	22.12	39	28.47	1	100.00	86	24.86	9.973 0.041
	No	98	47.12	45	32.85	0	0.00	143	41.33	
	Unsure	64	30.77	53	38.69	0	0.00	117	33.82	
	Total	208	100.00	137	100.00	1	100.00	346	100.00	
Question	Response	Men		Women		Total				
		N	%	N	%	N	%			Pearson X^2^ and *p* value
Do you eat the same amount of daily calories during off-season and in-season	Yes	42	30.00	53	22.08	95	25.00			6.561 0.038
	No	61	43.57	94	39.17	155	40.79			
	Unsure	37	26.43	93	38.75	130	34.21			
	Total	140	100.00	240	100.00	380	100.00			

### Men vs. Women athletes

When comparing men’s and women’s dietary habits, more men reported trying to gain weight (25.00 % vs. 4.17 %, *X* ^2^ = 40.037, *p* = 0.001), consumed fast food with greater frequency during a 7-day period (61.97 % vs. 53.33 %, *X* ^2^ = 15.021, *p* = 0.02) and consumed restaurant prepared meals with greater frequency during a 7-day week (80.85 % vs. 70.09 %, *X* ^2^ = 14.522, *p* = 0.013) (Table 5). Men were also less likely to prepare their own meals compared to women with a higher percentage replying “never” when asked how many times they prepare breakfast, lunch, or dinner, within a 7-day week (20.71 % vs. 9.66 %, *X* ^2^ = 15.746, *p* = 0.015).

**Table 5:** General Eating Habits

Question	Response	AthSD		AthNoSD		Unanswered		Total		
		N	%	N	%	N	%	N	%	Pearson X^2^ and *p* value
In one typical weekday (Monday-Friday), how many times do you eat beans/nuts/soy	Never	32	15.46	28	20.59	0	0.00	60	17.44	38.034 0.001
	1-2	112	54.11	55	40.44	0	0.00	167	48.55	
	3–4	28	13.53	36	26.47	0	0.00	64	18.60	
	5–6	21	10.14	6	4.41	0	0.00	27	7.85	
	7–8	10	4.83	5	3.68	1	100.00	16	4.65	
	9–10	2	0.97	2	1.47	0	0.00	4	1.16	
	>10	2	0.97	4	2.94	0	0.00	6	1.74	
	Total	207	100.00	136	100.00	1	100.00	344	100.00	
		N	%	N	%	N	%	N	%	Pearson X^2^ and *p* value
In one typical weekday (Monday-Friday), how many times do you eat sweets/dessert	Never	25	12.08	17	12.32	0	0.00	42	12.14	74.592 0.001
	1–2	102	49.28	63	45.65	0	0.00	165	47.69	
	3–4	47	22.71	29	21.01	0	0.00	76	21.97	
	5–6	22	10.63	12	8.70	0	0.00	34	9.83	
	7–8	7	3.38	10	7.25	0	0.00	17	4.91	
	9–10	2	0.97	2	1.45	1	100.00	5	1.45	
	>10	2	0.97	5	3.62	0	0.00	7	2.02	
	Total	207	100.00	138	100.00	1	100.00	346	100.00	
Question	Response	Men		Women		Total				
		N	%	N	%	N	%			Pearson X^2^ and *p* value
Are you a vegetarian	Yes	0	0.00	8	3.33	8	2.10			4.801 0.028
	No	141	100.00	232	96.67	373	97.90			
	Total	141	100.00	240	100.00	381	100.00			
		N	%	N	%	N	%			Pearson X^2^ and *p* value
Do you eat meat (Ex: beef, pork)	Yes	140	99.29	223	92.92	363	95.28			8.017 0.005
	No	1	0.71	17	7.08	18	4.72			
	Total	141	100.00	240	100.00	381	100.00			
		N	%	N	%	N	%			Pearson X^2^ and *p* value
Are you trying to gain weight	Yes	35	25.00	10	4.17	45	11.84			40.037 0.001
	No	101	72.14	228	95.00	329	86.58			
	N/A	4	2.86	2	0.83	6	1.58			
	Total	140	100.00	240	100.00	380	100.00			
		N	%	N	%	N	%			Pearson X^2^ and *p* value
Throughout a 7-day week, how many times do you eat fast food	Never	54	38.03	112	46.67	166	43.46			15.021 0.020
	1–2	55	38.73	100	41.67	155	40.58			
	3–4	22	15.49	14	5.83	36	9.42			
	5–6	8	5.63	7	2.92	15	3.93			
	7–8	2	1.41	6	2.50	8	2.09			
	9–10	1	0.70	0	0.00	1	0.26			
	>10	0	0.00	1	0.42	1	0.26			
	Total	142	100.00	240	100.00	382	100.00			
		N	%	N	%	N	%			Pearson X^2^ and *p* value
Throughout a 7-day week, how many times do you eat at a restaurant that prepares and delivers food to your table, or order take-out food (non-fast food)	Never	27	19.15	70	29.91	97	25.87			14.522 0.013
	1–2	80	56.74	129	55.13	209	55.73			
	3–4	24	17.02	32	13.68	56	14.93			
	5–6	6	4.26	3	1.28	9	2.40			
	7–8	2	1.42	0	0.00	2	0.53			
	9–10	2	1.42	0	0.00	2	0.53			
	Total	141	100.00	234	100.00	375	100.00			
		N	%	N	%	N	%			Pearson X^2^ and *p* value
Throughout a 7-day week, how many times do you prepare breakfast/lunch/dinner	Never	29	20.71	23	9.66	52	13.76			15.746 0.015
	1–2	34	24.29	58	24.37	92	24.34			
	3–4	28	20.00	45	18.91	73	19.31			
	5–6	19	13.57	26	10.92	45	11.90			
	7–8	7	5.00	29	12.18	36	9.52			
	9–10	3	2.14	10	4.20	13	3.44			
	>10	20	14.29	47	19.75	67	17.72			
	Total	140	100.00	238	100.00	378	100.00			
		N	%	N	%	N	%			Pearson X^2^ and *p* value
Throughout a 7-day week, how many times do you eat at an on campus food service	Never	23	16.20	56	23.43	79	20.73			14.727 0.022
	1–2	27	19.01	61	25.52	88	23.10			
	3–4	21	14.79	32	13.39	53	13.91			
	5–6	19	13.38	32	13.39	51	13.39			
	7–8	12	8.45	24	10.04	36	9.45			
	9–10	7	4.93	10	4.18	17	4.46			
	>10	33	23.24	24	10.04	57	14.96			
	Total	142	100.00	239	100.00	381	100.00			
		N	%	N	%	N	%			Pearson X^2^ and *p* value
In one typical weekday (Monday-Friday), how many times do you eat beef/chicken/fish/eggs	Never	0	0.00	5	2.09	5	1.32			23.813 0.001
	1–2	31	21.99	97	40.59	128	33.68			
	3–4	32	22.70	55	23.01	87	22.89			
	5–6	33	23.40	26	10.88	59	15.53			
	7–8	23	16.31	32	13.39	55	14.47			
	9–10	10	7.09	11	4.60	21	5.53			
	>10	12	8.51	13	5.44	25	6.58			
	Total	141	100.00	239	100.00	380	100.00			
		N	%	N	%	N	%			Pearson X^2^ and *p* value
In one typical weekday (Monday-Friday), how many times do you eat dairy products	Never	1	0.70	12	5.02	13	3.41			14.370 0.026
	1–2	32	22.54	67	28.03	99	25.98			
	3–4	44	30.99	72	30.13	116	30.45			
	5–6	28	19.72	32	13.39	60	15.75			
	7–8	14	9.86	31	12.97	45	11.81			
	9–10	13	9.15	8	3.35	21	5.51			
	>10	10	7.04	17	7.11	27	7.09			
	Total	142	100.00	239	100.00	381	100.00			

Hydration habits (Table 6) of the men showed they were more likely than women to drink juice during the week (90.78 % vs. 62.45 % *X* ^2^ = 38.353, *p* = 0.001) and on weekends (85.71 % vs. 60.83 %, *X* ^2^ = 31.542, *p* = 0.001), sports drinks (week: 97.14 % vs. 82.43 %, *X* ^2^ = 36.537, *p* = 0.001; weekend: 88.57 % vs. 60.42 %, *X* ^2^ = 38.522, *p* = 0.001), and energy drinks (week: 22.46 % vs. 5.86 %, *X* ^2^ = 26.017, *p* = 0.001; weekend: 21.74 % vs. 4.58 %, *X* ^2^ = 28.389, *p* = 0.001). The men also drank more soda on the weekend (54.61 % vs. 40.00 %, *X* ^2^ = 8.423, *p* = 0.038). Women consumed water more frequently one hour prior to practice, training, and competition (74.17 % vs. 53.85 %, *X* ^2^ = 33.022, *p* = 0.001). Survey questions about alcohol consumption showed a higher weekly alcohol intake among the men (47.55 % vs. 27.50 %, *X* ^2^ = 24.725, *p* = 0.001). Additionally, the men were more likely to drink alcohol during the competitive season (24.82 % vs. 14.23 %, *X* ^2^ = 6.701, *p* = 0.01). Based upon our results, 23.94 % of men and 10.00 % of women could be at risk for binge drinking.

**Table 6:** Hydration

Question	Response	AthSD		AthNoSD		Unanswered		Total		
		N	%	N	%	N	%	N	%	Pearson X^2^ and *p* value
In one typical weekday (Monday-Friday), how many times do you drink a serving of energy drinks	Never	178	86.41	123	90.44	0	0.00	301	87.76	175.122 0.001
	1–2	21	10.19	9	6.62	0	0.00	30	8.75	
	3–4	4	1.94	3	2.21	0	0.00	7	2.04	
	5–6	0	0.00	1	0.74	1	100.00	2	0.58	
	7–8	2	0.97	0	0.00	0	0.00	2	0.58	
	>10	1	0.49	0	0.00	0	0.00	1	0.29	
	Total	206	100.00	136	100.00	1	100.00	343	100.00	
		N	%	N	%	N	%	N	%	Pearson X^2^ and *p* value
In one typical weekend day (Saturday/Sunday), how many times do you drink a serving of juice (8 oz.)	Never	67	32.21	37	27.01	0	0.00	104	30.06	24.561 0.017
	1–2	79	37.98	62	45.26	0	0.00	141	40.75	
	3–4	44	21.15	23	16.79	0	0.00	67	19.36	
	5–6	10	4.81	7	5.11	1	100.00	18	5.20	
	7–8	6	2.88	3	2.19	0	0.00	9	2.60	
	9–10	1	0.48	4	2.92	0	0.00	5	1.45	
	>10	1	0.48	1	0.73	0	0.00	2	0.58	
	Total	208	100.00	137	100.00	1	100.00	346	100.00	
		N	%	N	%	N	%	N	%	Pearson X^2^ and *p* value
In one typical weekend day (Saturday/Sunday), how many times do you drink a serving of energy drinks	Never	181	87.44	124	91.18	0	0.00	305	88.66	174.541 0.001
	1–2	22	10.63	8	5.88	0	0.00	30	8.72	
	3–4	4	1.93	3	2.21	0	0.00	7	2.03	
	5–6	0	0.00	1	0.74	1	100.00	2	0.58	
	Total	207	100.00	136	100.00	1	100.00	344	100.00	
Question	Response	Men		Women		Total				
		N	%	N	%	N	%			Pearson X^2^ and *p* value
In one typical weekday (Monday-Friday), how many times do you drink a serving of juice (8 oz.)	Never	13	9.22	89	37.55	102	26.98			38.353 0.001
	1–2	57	40.43	77	32.49	134	35.45			
	3–4	31	21.99	34	14.35	65	17.20			
	5–6	21	14.89	22	9.28	43	11.38			
	7–8	11	7.80	9	3.80	20	5.29			
	9–10	4	2.84	3	1.27	7	1.85			
	>10	4	2.84	3	1.27	7	1.85			
	Total	141	100.00	237	100.00	378	100.00			
		N	%	N	%	N	%			Pearson X^2^ and *p* value
In one typical weekend day (Saturday/Sunday), how many times do you drink a serving of juice (8 oz.)	Never	20	14.29	94	39.17	114	30.00			31.542 0.001
	1–2	65	46.43	88	36.67	153	40.26			
	3–4	34	24.29	41	17.08	75	19.74			
	5–6	13	9.29	9	3.75	22	5.79			
	7–8	6	4.29	3	1.25	9	2.37			
	9–10	1	0.71	4	1.67	5	1.32			
	>10	1	0.71	1	0.42	2	0.53			
	Total	140	100.00	240	100.00	380	100.00			
		N	%	N	%	N	%			Pearson X^2^ and *p* value
In one typical weekday (Monday-Friday), how many times do you drink a serving of a sport drink (8 oz.)	Never	4	2.86	42	17.57	46	12.14			36.537 0.001
	1–2	40	28.57	78	32.64	118	31.13			
	3–4	26	18.57	52	21.76	78	20.58			
	5–6	30	21.43	28	11.72	58	15.30			
	7–8	10	7.14	20	8.37	30	7.92			
	9–10	13	9.29	12	5.02	25	6.60			
	>10	17	12.14	7	2.93	24	6.33			
	Total	140	100.00	239	100.00	379	100.00			
		N	%	N	%	N	%			Pearson X^2^ and *p* value
In one typical weekend day (Saturday/Sunday), how many times do you drink a serving of a sports drink (8 oz.)	Never	16	11.43	95	39.58	111	29.21			38.522 0.001
	1–2	56	40.00	78	32.50	134	35.26			
	3–4	34	24.29	38	15.83	72	18.95			
	5–6	20	14.29	15	6.25	35	9.21			
	7–8	7	5.00	10	4.17	17	4.47			
	9–10	3	2.14	2	0.83	5	1.32			
	>10	4	2.86	2	0.83	6	1.58			
	Total	140	100.00	240	100.00	380	100.00			
		N	%	N	%	N	%			Pearson X^2^ and *p* value
In one typical weekday (Monday-Friday), how many times do you drink a serving of hot or iced coffee (8 oz.)	Never	87	63.50	114	47.70	201	53.46			16.970 0.009
	1–2	34	24.82	71	29.71	105	27.93			
	3–4	8	5.84	25	10.46	33	8.78			
	5–6	3	2.19	12	5.02	15	3.99			
	7–8	3	2.19	11	4.60	14	3.72			
	9–10	0	0.00	6	2.51	6	1.60			
	>10	2	1.46	0	0.00	2	0.53			
	Total	137	100.00	239	100.00	376	100.00			
		N	%	N	%	N	%			Pearson X^2^ and *p* value
In one typical weekend day (Saturday/Sunday), how many times do you drink a serving of hot or iced coffee (8 oz.)	Never	98	70.50	140	58.33	238	62.80			13.883 0.031
	1–2	31	22.30	72	30.00	103	27.18			
	3–4	4	2.88	22	9.17	26	6.86			
	5–6	2	1.44	5	2.08	7	1.85			
	7–8	2	1.44	1	0.42	3	0.79			
	9–10	1	0.72	0	0.00	1	0.26			
	>10	1	0.72	0	0.00	1	0.26			
	Total	139	100.00	240	100.00	379	100.00			
		N	%	N	%	N	%			Pearson X^2^ and *p* value
In one typical weekday (Monday-Friday), how many times do you drink a serving of energy drinks	Never	107	77.54	225	94.14	332	88.06			26.017 0.001
	1–2	21	15.22	11	4.60	32	8.49			
	3–4	4	2.90	3	1.26	7	1.86			
	5–6	3	2.17	0	0.00	3	0.80			
	7–8	2	1.45	0	0.00	2	0.53			
	>10	1	0.72	0	0.00	1	0.27			
	Total	138	100.00	239	100.00	377	100.00			
		N	%	N	%	N	%			Pearson X^2^ and *p* value
In one typical weekend day (Saturday/Sunday), how many times do you drink a serving of an energy drink	Never	108	78.26	229	95.42	337	89.15			28.389 0.001
	1–2	23	16.67	8	3.33	31	8.20			
	3–4	4	2.90	3	1.25	7	1.85			
	5–6	3	2.17	0	0.00	3	0.79			
		N	%	N	%	N	%			Pearson X^2^ and *p* value
In one typical weekend day (Saturday/Sunday), how many times do you drink a serving of soda	Never	64	45.39	144	60.00	208	54.59			8.423 0.038
	1–2	63	44.68	74	30.83	137	35.96			
	3–4	11	7.80	16	6.67	27	7.09			
	5–6	3	2.13	6	2.50	9	2.36			
	Total	141	100.00	240	100.00	381	100.00			
		N	%	N	%	N	%			Pearson X^2^ and *p* value
In one typical weekday (Monday-Friday), how many times do you drink a serving of hot or iced tea (8 oz.)	Never	98	71.53	140	58.33	238	63.13			17.056 0.009
	1–2	26	18.98	57	23.75	83	22.02			
	3–4	7	5.11	23	9.58	30	7.96			
	5–6	6	4.38	4	1.67	10	2.65			
	7–8	0	0.00	11	4.58	11	2.92			
	9–10	0	0.00	3	1.25	3	0.80			
	>10	0	0.00	2	0.83	2	0.53			
	Total	137	100.00	240	100.00	377	100.00			
		N	%	N	%	N	%			Pearson X^2^ and *p* value
How many water servings do you normally drink an hour prior to practice/training/competition	0	4	2.80	11	4.58	15	3.92			33.022 0.001
	1–2	77	53.85	178	74.17	255	66.58			
	3–4	41	28.67	41	17.08	82	21.41			
	5–6	16	11.19	4	1.67	20	5.22			
	7–8	3	2.10	3	1.25	6	1.57			
	9–10	0	0.00	3	1.25	3	0.78			
	>10	2	1.40	0	0.00	2	0.52			
	Total	143	100.00	240	100.00	383	100.00			
		N	%	N	%	N	%			Pearson X^2^ and *p* value
How many water servings do you normally drink within two hours after practice/training/competition	0	3	2.10	2	0.83	5	1.31			19.804 0.003
	1–2	37	25.87	113	47.08	150	39.16			
	3–4	67	46.85	81	33.75	148	38.64			
	5–6	19	13.29	30	12.50	49	12.79			
	7–8	9	6.29	6	2.50	15	3.92			
	9–10	5	3.50	5	2.08	10	2.61			
	>10	3	2.10	3	1.25	6	1.57			
	Total	143	100.00	240	100.00	383	100.00			
		N	%	N	%	N	%			Pearson X^2^ and *p* value
Throughout a 7-day week about how many days do you drink alcohol	0	75	52.45	174	72.50	249	65.01			24.725 0.001
	1–2	51	35.66	61	25.42	112	29.24			
	3–4	14	9.79	3	1.25	17	4.44			
	5–6	2	1.40	1	0.42	3	0.78			
	7	1	0.70	1	0.42	2	0.52			
	Total	143	100.00	240	100.00	383	100.00			
		N	%	N	%	N	%			Pearson X^2^ and *p* value
How many alcoholic beverages do you usually consume in one sitting	0	65	45.77	154	64.17	219	57.33			28.560 0.001
	1–2	20	14.08	33	13.75	53	13.87			
	3–4	23	16.20	29	12.08	52	13.61			
	5–6	13	9.15	17	7.08	30	7.85			
	7–8	10	7.04	5	2.08	15	3.93			
	9–10	6	4.23	1	0.42	7	1.83			
	11–12	2	1.41	0	0.00	2	0.52			
	13–14	3	2.11	0	0.00	3	0.79			
	15–16	0	0.00	1	0.42	1	0.26			
	Total	142	100.00	240	100.00	382	100.00			
		N	%	N	%	N	%			Pearson X^2^ and *p* value
Do you drink alcohol during your competitive season	Yes	35	24.82	34	14.23	69	18.16			6.701 0.010
	No	106	75.18	205	85.77	311	81.84			
	Total	141	100.00	239	100.00	380	100.00			

Post-workout nutrition data (Table 2) indicate that following practices, training, or competitions, men were more likely than women to consume rice or pasta as their primary carbohydrate source (69.23 % vs. 52.72 %, *X* ^2^ = 10.076, *p* = 0.002). While on team trips (Table 3) away from campus, the men reported more fast food being provided prior to practice or competitions (19.85 % vs. 9.91 %, *X* ^2^ = 7.214, *p* = 0.007).

Women were more likely than the men to prepare meals (Table 5) during a 7-day week (90.34 % vs. 79.29 %, *X* ^2^ = 15.746, *p* = 0.015), eat breakfast (Table 7) 7 days a week (57.56 % vs. 36.62 %, *X* ^2^ = 27.691, *p* = 0.001), as well as eat breakfast before all practices (65.83 % vs. 55.24 %, *X* ^2^ = 4.259, *p* = 0.039) and training/lifting sessions (65.00 % vs. 51.75 %, *X* ^2^ = 6.560, *p* = 0.01). Coffee consumption (Table 6) was higher in women (week: 52.30 % vs. 36.5 %, *X* ^2^ = 16.970, *p* = 0.009; weekend: 41.67 % vs. 29.5 %, *X* ^2^ = 13.883, *p* = 0.031) and tea consumption was higher in women (week: 41.67 % vs. 28.47 %, *X* ^2^ = 17.056, *p* = 0.009).

**Table 7:** Breakfast

Question	Response	AthSD		AthNoSD		Unanswered		Total		
		N	%	N	%	N	%	N	%	Pearson X^2^ and *p* value
Do you eat breakfast before all competitions	Yes	184	88.89	124	89.86	0	0.00	308	89.02	8.208 0.017
	No	23	11.11	14	10.14	1	100.00	38	10.98	
	Total	207	100.00	138	100.00	1	100.00	346	100.00	
Question	Response	Men		Women		Total				
		N	%	N	%	N	%			Pearson X^2^ and *p* value
How many days a week do you eat breakfast	0	5	3.52	3	1.26	8	2.11			27.691 0.001
	1	1	0.70	5	2.10	6	1.58			
	2	13	9.15	20	8.40	33	8.68			
	3	20	14.08	11	4.62	31	8.16			
	4	13	9.15	11	4.62	24	6.32			
	5	28	19.72	29	12.18	57	15.00			
	6	10	7.04	22	9.24	32	8.42			
	7	52	36.62	137	57.56	189	49.74			
	Total	142	100.00	238	100.00	380	100.00			
		N	%	N	%	N	%			Pearson X^2^ and *p* value
What time of day do you normally eat breakfast	I don’t eat breakfast	7	4.93	6	2.51	13	3.41			28.418 0.001
	5:00–7:00 AM	8	5.63	45	18.83	53	13.91			
	7:00 AM–9:00 AM	70	49.30	134	56.07	204	53.54			
	9:00–11:00 AM	56	39.44	47	19.67	103	27.03			
	11:00 AM–1:00 PM	1	0.70	7	2.93	8	2.10			
	Total	142	100.00	239	100.00	381	100.00			
		N	%	N	%	N	%			Pearson X^2^ and *p* value
Do you eat breakfast before all practices	Yes	79	55.24	158	65.83	237	61.88			4.259 0.039
	No	64	44.76	82	34.17	146	38.12			
	Total	143	100.00	240	100.00	383	100.00			
		N	%	N	%	N	%			Pearson X^2^ and *p* value
Do you eat breakfast before all training/lifting sessions	Yes	74	51.75	156	65.00	230	60.05			6.560 0.010
	No	69	48.25	84	35.00	153	39.95			
	Total	143	100.00	240	100.00	383	100.00			
		N	%	N	%	N	%			Pearson X^2^ and *p* value
Do you eat breakfast before all competitions	Yes	121	84.62	218	91.60	339	88.98			4.439 0.035
	No	22	15.38	20	8.40	42	11.02			
	Total	143	100.00	238	100.00	381	100.00			

Women were more likely than men to have post-workout nutrition options provided (57.14 % vs. 45.19 %, *X* ^2^ = 4.888, *p* = 0.027) and preferential post-workout carbohydrate sources following practices, training, or competitions for women was fruit (33.89 % vs. 23.08 %, *X* ^2^ = 4.998, *p* = 0.025) (Table 2). During team trips, women were more likely to receive school-provided boxed breakfast, lunch, or dinner (18.10 % vs. 8.82 %, *X* ^2^ = 5.897, *p* = 0.015) and to have food available after games or between same-day competitions (90.48 % vs. 79.71 %, *X* ^2^ = 12.370, *p* = 0.002) (Table 3). Further, the women were more likely than the men to be unsure of whether or not they ate the same amount of daily calories during the off-season and in-season (22.08 % vs. 30.00 %, *X* ^2^ = 6.561, *p* = 0.038) (Table 4).

## Discussion

The first aim of the present study was to examine differences that having access to a full time SD as the primary nutrition information source may have on the dietary habits and practices of NCAA Division I athletes. The second aim was to assess differences in dietary habits and practices between men and women collegiate athletes.

Valliant et al. [1] utilized an RD to deliver nutritional interventions to eleven NCAA Division I volleyball athletes. The aforementioned women athletes received individualized dietary counseling once a month for four months, which resulted in significant improvements in energy intake, macronutrient intake, and knowledge of sports nutrition [1]. The aforementioned data are supported by our findings, which indicate access to an SD can help athletes, who seek counsel, to improve the application of performance nutrition principals. However, limited resources and time constraints may prevent SDs from providing intensive education to all athletes and/or teams at their specific institution.

Convenience has been identified as the biggest factor as to why college students choose to consume fast foods [29, 30]. However, SDs can play an important role in assisting both individual athletes and sport teams with establishing healthy food options that are also convenient. Data from the current study showed that 13.69 % of athletes consumed fast foods prior to practice or competition, and that consumption was more frequent among men. These data are in accordance with previously published surveys showing that 15 % of athletes received fast food meals, which are typically of poor nutrient quality, during team-sponsored trips [31], and that men college students were more likely to consume fast foods than women college students [32]. Additionally, athletes in our study who used a SD as their primary source of nutrition information were 49.36 % less likely to consume fast food before a practice or competition. Further, our data also indicate a lower overall intake of fast foods than what has been previously reported [22, 29]. Providing convenient healthy food items to athletes may help to minimize fast food consumption.

With regard to alcohol consumption, the National Institute on Alcohol Abuse and Alcoholism defines binge drinking as “a pattern of drinking that brings blood alcohol concentration (BAC) levels to 0.08 g/dL… [typically occurring] after 4 drinks for women and 5 drinks for men—in about 2 h” [33]. In the current study, a cutoff value of consuming 5 drinks or more in one sitting was used for all survey respondents. Survey question design prevented our using the common binge drinking definition of “4 drinks or more” for the women. Thus, the actual rate of potential women binge drinkers is likely to be higher, with a maximum possible upper limit of 22.08 %. In agreement with a recent NCAA report on substance use trends among NCAA college student-athletes [34], the majority of surveyed athletes who reported alcohol consumption did so ≤ two days per week (83.58 %). Further, the NCAA study found that 49 % of athletes who reported consuming alcohol consumed five or more drinks in one sitting [34], while our data showed a substantially lower rate of 35.58 % among surveyed athletes. Alcohol consumption during the competitive season may worsen performance. Excessive alcohol intake in the recovery period may cause or exacerbate dehydration due to its diuretic effects in addition to blunting the overall recovery process [4]. Our survey results suggest only a satisfactory compliance with the recommendation to minimize or avoid alcohol intake in order to maximize performance [4].

Athletes commonly acknowledge using non-SDs (e.g., strength coach, athletic trainer) as their primary nutrition information sources [11, 13, 35, 36]. Torres-McGehee et al. [11] found that 58.2 % of NCAA players, sport coaches, strength coaches, and athletic trainers across all divisions (I, II and III) had access to a RD with only 21.9 % having a full-time RD designated to work specifically with athletes. Athletes not only reported seeking nutritional advice from strength coaches (15 %), parents (12 %), and athletic trainers (10 %) [11], but using these individuals as primary nutrition information sources [37]. Although athletic trainers, coaches, and strength staff may have a strong knowledge base in regard to nutritional recommendations for athletes [31], not all of them fall into this category. Torres-McGehee [11] found that approximately 30 % of athletic trainers and 17 % of strength coaches had inadequate nutrition knowledge. Similarly, Rockwell et al. [31] found that coaches and athletic trainers often had a poor understanding of energy requirements, macronutrient, and micronutrient needs of athletes. Future research is warranted that examines the effects an SD has on nutritional knowledge of strength coaches, athletic trainers, and team coaches.

Unfamiliarity with the SD’s availability to the athletes is an additional obstacle to delivering sports nutrition information. Burns et al. observed that 23.5 % of athletes reported not knowing a dietitian was accessible [35]. Similarly, while all athletes surveyed in the present study had access to a SD, 39.7 % reported using a non-SD source as their primary nutrition information resource. Without guidance from an evidence-based practitioner, athletes may be prone to taking on dietary habits detrimental to their performance goals [38].

While an athlete might not meet directly with a SD, he or she may still benefit from the presence of a SD employed within an athletic program. SDs often work collaboratively with athletic staff to inform them of best performance nutrition practices. Athletic staff can then reinforce these positive behaviors among their athletes. In the present study, dietary habits, which assist in promoting optimal performance, reported by all athletes, regardless of primary nutrition information source (i.e., SD or no-SD), included consuming breakfast before all competitions (89 %), knowing what their primary post-practice, training, or competition carbohydrate and protein sources were (93.19 % and 91.36 %, respectively), and eating within one hour of practice (75.80 %). Athletes also reported a lower incidence of supplement use (44 %) than that reported in previous studies of athletes [23, 35, 39]. Supplement use among NCAA athletes is of particular concern, as the ingestion of one that contains impermissible substances can result in temporary or permanent loss of eligibility to play [6]. As previous studies have reported, athlete knowledge of dietary supplement use [1, 11, 23] and safety [1, 23] are low. It is recommended that SDs work to inform athletes and athletic staff of the efficacy and risks associated with supplement use.

Results of concern from the current study indicate future target areas for SDs to address: 50.93 % reported experiencing hunger during training, practice, or competition, 30.68 % reported experiencing negative effects from dehydration while a university athlete, and 18.16 % reported alcohol consumption during the competitive season. Experiencing hunger during training, practice, or competition is suggestive of low energy availability or poor pre-exercise fueling compliance, which increases the risk for reduced performance, muscle protein degradation, and an impaired ability to recover or augment training adaptations [4]. Half of all student-athletes reported these feelings of hunger indicating a lack of adherence to pre-exercise meal recommendations [27]. One-third of athletes reported suffering from dehydration at one point or another during their career as a university athlete, although frequency of these occurrences was not measured. Nevertheless, this indicates only a satisfactory adherence to basic hydration principals [40]. Athletes should aim to replace lost fluids so that the total body fluid loss is <2 % of body weight as to minimize any impairments to performance [4].

Collegiate athletes who are not receiving evidence-based information may also have a lack of basic nutritional knowledge and understanding of how nutrition can be used to improve performance [11, 21, 26, 36, 41]. Jacobsen et al. [36] found that, of the 320 college Division I men and women athletes surveyed, 30 % were able to identify the recommended carbohydrate intake and only 3 % were able to identify the correct protein intake. Similarly, Torres-McGehee et al. [11] reported that surveyed athletes possessed a limited knowledge of sound nutritional practices as was evident by the percentage of questions answered correctly that pertained to: weight management (66 %), micronutrient and macronutrient intake (55 %), hydration (54 %), supplements and performance (52 %), and eating disorders (47 %). It should be noted that athletes very likely have higher energy requirements than those advised by the recommended daily allowance (RDA) and; therefore, nutrient intakes should be higher than RDA values [42]. Several studies have assessed actual nutrient intakes of collegiate athletes and the majority has found that athletes had energy and micronutrient intakes below or similar to RDA values [20, 21, 23, 43] suggesting athletes do not follow dietary recommendations. A lack of proper nutritional intake could potentially lead to negative health outcomes, unfavorable changes in body composition, and decreases in performance. Prior research has shown that completing a college nutrition course may help student athletes improve nutrition knowledge [12, 44]. Nutrition courses or seminars for athletes taught by a SD may be a viable solution for universities with financial constraints.

While the strengths of our study included a large sample size of men and women athletes and a high survey response rate, we acknowledge some limitations. Such limitations may include a survey’s susceptibility to recall bias and under- or over-reporting by subjects. Additionally, a full time SD was employed at both participating universities, allowing for the possibility that effects observed could be influenced by the SD’s general outreach efforts (e.g., educational bulletin boards, athletics/sport nutrition website content) and interactions with the athletes and athletic staff (e.g., social media, sport nutrition lectures, blogs).

The amount and availability of team funds may have an effect on an athlete’s access to supplements and food preparation. It is likely that better funded teams (e.g., revenue generating sports) may be in a more favorable position to provide more comprehensive sports nutrition professional services.

Therefore, it is recommended that future research examine the effect of funding on said services provided to athletes.

## Conclusion

Positive effects of the use of a SD as the primary nutrition information source were observed in post-workout nutrition habits, nutrition during team trips, and nutrient periodization. Significant differences in survey responses between genders were noted in alcohol use, frequency and timing of fast food consumption, and uncertainty or inaccurate understanding of nutrient periodization. Collegiate athletes and athletic support staff need access to and training in the recommended evidence-based performance nutrition principals to ensure quality information is being provided. Cooperative working relationships between athletic staff and SDs can ensure such information is being delivered to the athlete; therefore, it is recommended that SDs promote themselves within their respective athletic departments in order to increase awareness of the services they can provide.
